# Haemorrhage Complicating Anticoagulant Therapy

**Published:** 1975

**Authors:** R. C. Bucknall, R. D. Eastham


### Haemorrhage Complicating Anticoagulant Therapy

R. C. Bucknall and R. D. Eastham

A significant incidence of haemorrhage as a complication of anticoagulant therapy has previously been reported, underlining the potential danger of this form of treatment. Fuller (1959) reported 194 episodes of haemorrhage in 105 patients, out of a total of 217 patients on anticoagulant therapy. In an assessment of long term oral anticoagulant therapy following myocardial infarction the Medical Research Council (1964) reported 80 episodes of haemorrhage during 5, 101 patient/month of treatment of 195 patients and of these episodes 16 were serious. Hazard et al (1967) reported ten cases of intracranial haemorrhage during a two and a half year period. Seventy-six haemorrhagic episodes were reported during the treatment of 507 patients during a five year period (Serradimigni, 1969) while Straub (1970) recorded 13 deaths from haemorrhage in 70 patients treated with anticoagulant therapy. Bleeding was detected during 263 (6.8%) out of 3,862 courses of anticoagulant therapy with an identifiable lesion responsible for bleeding in half of these and death attributed to haemorrhage occurred in four cases (Coon et al, 1974).

Inadequate attention has been paid to the mechanisms of haemorrhage in patients receiving anticoagulant therapy. The object of this prospective study was to determine the cause of haemorrhage in each case and outline ways in which this potentially serious complication of this frequently used treatment could be prevented.

### METHOD

Patients receiving anticoagulant therapy were studied over a two year period.

They were receiving heparin and/or warfarin or phenindione. Anticoagulant dosage was controlled by means of the plasma activated partial thromboplastin time (APTT) in preference to the prothrombin ratio (PR) since the APTT can be used to control both heparin and oral anticoagulant dosage (Eastham, 1968). Heparin was administered via a constant intravenous infusion line at approximately 10,000 units per six hours, the rate of infusion being adjusted to maintain the APTT in the range 50-100 seconds, using a graph relating the drip rate to the required APTT value. Warfarin (or phenindione) dosage was adjusted to maintain the APTT in the range of 50-70 seconds. In patients who were started on heparin and warfarin therapy simultaneously, heparin was discontinued four days later and blood collected for measurement of APTT an hour after stopping the intravenous drip. This reading indicated whether the warfarin was acting effectively.

The clinical conditions treated by anticoagulant therapy are shown in table I.

### RESULTS

During the period of two years 50 haemorrhagic episodes were recorded, 44 patients each bleeding once, while three patients each had two bleeding episodes. At any time approximately 12 inpatients and 100 outpatients were receiving anticoagulant therapy.

### Time of onset of bleeding during anticoagulant therapy.

Eight patients bled during the first week of treatment, five while being treated with heparin alone and three during treatment with heparin and warfarin. From the end of the first week until the end of the first month a further ten bleeding episodes occurred, one in an inpatient and nine in outpatients, a total of 18 episodes during the first four weeks (36% of the total bleeds).

A further eight bleeding episodes were recorded in the period from the beginning of the second month until the end of the third month, so that more than half of the haemorrhages had occurred in patients during the first 12 weeks of treatment. Three episodes occurred during the fourth and fifth month, a further three during the seventh to twelfth month of treatment and 18 patients suffered from haemorrhage after receiving Warfarin (15 cases) or phenindione (three cases) for more than one year.

### Type of anticoagulant used and Laboratory Control of Therapy.

At the time of bleeding 38 patients were being treated with warfarin, four with phenindione, five with heparin and three with heparin and warfarin. From the end of the first week of treatment none of the patients studied were receiving heparin.

The APTT and PR were checked before anticoagulant therapy was started in 15 patients to exclude any unsuspected bleeding tendency and a routine blood count was also carried out in all patients when thrombocytopaenia was excluded. In 35 patients no initial checks of the APTT and PR were carried out before treatment and in six of these patients the APTT exceeded the upper limit of the therapeutic range at the time of bleeding.

The results from the five patients who bled while being treated with heparin only showed APTT readings of 100 seconds in one case and of more than 100 seconds in four cases. In three patients treated with heparin plus warfarin, at the time of haemorrhage, two PR results fell within the therapeutic range (1.5—2.1) and one PR result exceeded this at 3.2, with APTT results of 89, 125 and more than 250 seconds respectively, all exceeding the therapeutic range for warfarin therapy of 50—70 seconds.

In eight cases of bleeding during treatment with warfarin only, the APTT was above the upper limit of the therapeutic range, even though the PR was within the therapeutic range. Conversely, in three patients the PR exceeded the upper limit of the therapeutic range, while the corresponding APTT values fell within the therapeutic range.

**Site and Severity of Haemorrhage.**

The various sites at which bleeding occurred are shown in table 2. In only two cases did bleeding occur from previously recognised pathological lesions, one a duodenal ulcer, the other a sinus of the urinary tract.

At their first attendance after discharge from in-patient care, most of the outpatients had received verbal and printed instructions, both to discontinue oral anticoagulant therapy and to notify their medical attendants following the development of bleeding into the skin or visible haematuria. It was hoped thereby to reduce the risk of haemorrhage into those anatomical sites associated with a significant morbidity or mortality. Out of the 50 cases studied only three patients suffered severe morbidity with retroperitoneal haemorrhage (two) and subdural haematoma (one) in a young patient who sustained a head injury while on warfarin. One death could have been partly attributed to anticoagulant therapy, as haemorrhage occurred into infarcted brain secondary to a middle cerebral artery occlusion.

**Age of patient and complicating medical conditions.**

Thirty three of the patients were over 50 years, and of these 22 were over 60 years old. A total of eight patients out of the 50 cases studied had coincidental conditions known to be associated with an increased risk of haemorrhage during anticoagulant therapy. One had alcoholic cirrhosis, four had congestive cardiac failure, one had thrombocytopaenia and two patients were receiving corticosteroids.

**Drug Interactions.**

Concurrent use of drugs which potentiate the action of warfarin were responsible for haemorrhage in eight cases. Treatment with antibiotics was associated with haemorrhage in four cases, with clofibrate in two cases, with phenylbutazone in one case and with amitryptyline (not previously recorded as potentiating warfarin action) in one case.

**Excess Dose of Anticoagulant.**

It appeared that an excessive dose of anticoagulant was the cause of haemorrhage in 14 cases. In four of these this occurred during the first week of treatment, two of the patients being treated with heparin alone, the other two with heparin and warfarin. Six further cases developed bleeding in the period from the beginning of the second week to the end of the second month of treatment, five being treated with warfarin and one with phenindione, all as outpatients. All six cases had been discharged from hospital on too high a dose of anticoagulant. However, one of these cases was found to have a depressed plasma fibrinogen concentration. Another patient was discharged from hospital on an apparently correct dose of warfarin, but unfortunately the APTT and PR results used prior to discharge to assess the proposed dosage were carried out on blood collected soon after the administration of 5 units of transfused cells, containing 300 ml. of trapped plasma. This resulted in a temporary misleading reduction in the APTT and PR results.

**Other Causes of Bleeding.**

In 17 cases no cause for haemorrhage was found. There was no way of knowing whether outpatients were taking their prescribed dose of oral anticoagulant correctly; nor was there any way of knowing whether any of them took their own additional remedies, even though they were all warned at their first attendance at the anticoagulant clinic to avoid aspirin and other drugs. In only two cases could the patient be blamed for the haemorrhage. In four instances it was found that the care of the patient was divided between at least three doctors and this was an important factor in overdosage with warfarin.

### DISCUSSION

Treatment with anticoagulants is potentially dangerous and a prospective study of 50 consecutive bleeding episodes, associated with anticoagulant therapy, was therefore carried out to see whether the standard of anticoagulant control could be improved. The incidence of such episodes in outpatients attending the hospital during a five year period was 103, representing one bleeding episode per 133 weeks of treatment (Eastham, 1972). Although the majority of these episodes were mild (bruising and macroscopic haematuria), such bleeding is potentially very dangerous. Of the present 50 bleeds investigated 33 were probably avoidable. In a previous study (1967—1971) it had been found that nearly one third of the episodes occurred during the first month and 46% occurred during the first two months of treatment (Eastham, 1972). A similar incidence is found in the present series in the same hospital group during the following two years, 36% occurring within the first month and 50% within the first three months. These results are better than in another series reported by Fuller (1959) in which 105 patients out of a group of 217 patients suffered 194 bleeding episodes, of which 24 were described as severe and in which 69 bleeds occurred in 55 of the patients during the first three months of anticoagulant therapy.

The results of this study suggest certain practical ways in which the incidence of haemorrhage might be reduced. Prior to anticoagulant therapy either singly with heparin or warfarin, or with the two drugs combined, the plasma APTT and PR should be checked to exclude deficiency in plasma coagulation factors (normal APTT and PR results, obtainable in a few minutes, exclude deficiency of plasma factors I, II, V, VII, VIII, IX, X, XI and XII). Such patients have usually had a recent full blood count when the presence of platelets can be noted on examination of a blood film. Clinical examination of the patient should exclude such conditions as cardiac failure, iatrogenic Cushing's disease, duodenal ulceration, cirrhosis etc.

In a recent study 50% of episodes of haemorrhage arose from an identifiable pathological lesion (Coon et al, 1974). In this present series only two patients bled from a known lesion, one from a duodenal ulcer and the other from a sinus related to the urinary tract. The same authors (Coon et al, 1974) considered that the risk increased after middle age and in this present study 22 (44%) of the patients who bled were over 60 years.

Although the prothrombin ratio is estimated on all blood samples taken for anticoagulant control, the activated partial thromboplastin time is used in addition for controlling the dosage of warfarin or phenindione, since it has been shown to provide a more reliable warning of the likelihood of haemorrhagic accidents (Eastham, 1968). The APTT is also used to control the intravenous infusion of heparin and should be measured daily for this purpose. The clotting time performed by junior hospital doctors at the patient's bedside is less accurate. The present study confirms the relative safety of heparin anticoagulation, which is used frequently for prophylaxis against thromboembolism following myocardial infarction and in the treatment of deep vein thrombosis and pulmonary embolism. In this context, however, it is worth noting that after venous thrombosis or trauma the APTT tends to be at the lower end of the normal range, or below it (Eastham and Morgan, 1964) and this is reflected in some resistance to the action of heparin. This resistance decreases with recovery, so that the daily requirement of heparin falls. It is therefore important that daily APTT estimations should be carried out so that the rate of heparin infusion can be adjusted to prevent either under treatment with the risk of further thrombosis or overtreatment with the risk of bleeding (Eastham, Perham and Pocock, 1972). Two patients in this series suffered haemorrhage as a result of this mechanism.

In eight cases the APTT exceeded the therapeutic range, while in three cases the APTT was within the normal range at the time of haemorrhage, with the PR within the therapeutic range. This gives some support for the original suggestion that the APTT is more useful for the control of oral anticoagulant therapy than the PR (Eastham, 1968).

When warfarin therapy is used, the patient should be discharged only when the APTT is steady on a regular warfarin dose, and ideally, alterations in warfarin dosage should not be made more often than every 48 hours, and should not be large, to avoid wide fluctuation in APTT or PR results. Follow up at the anticoagulant clinic should not be later than one week after discharge from hospital. If possible, drugs which depress warfarin activity so that increased activity follows their withdrawal (e.g. barbiturate hypnotics) should either be discontinued many days before discharge or continued after discharge. Similarly, treatment with drugs which potentiate the action of warfarin (e.g. clofibrate) should not be initiated on discharge. The patient should be given a list of the many drugs which either potentiate, or depress, warfarin activity and the hospital doctors, haematologist and general practitioner, should inform each other of any new drug therapy or discontinuation of previous therapy.

A quarter of a century ago Askey and Cherry (1950) stated that safe effective long term anticoagulant therapy depends on a triad comprising the vigilant physician, the co-operative patient and the reliable laboratory. In this hospital there is a six-monthly lecture on anticoagulation therapy for all junior or hospital staff. This lecture incorporates special information on drug interactions, heparin sensitivity and the importance of taking into account the age of the patient and other pathological conditions, including haematological disorders. Patients are informed of the nature of the treatment and warned about the significance of bruising and haematuria, and are given a small booklet to reinforce this information. The first attendance is arranged to be only a few days after discharge from hospital. Communication between hospital doctor and general practitioner is good and, in addition the consultant haematologist has a special interest in anticoagulant therapy control. It is therefore disappointing that approximately 25 bleeding episodes should occur each year, even though 50% of these episodes could be considered mild (bruising and macroscopic haematuria).

The knowledge of how such haemorrhagic episodes occur is of no great value, unless it is used positively to reduce the future incidence of anticoagulation overdosage.

**Summary**

A prospective study of patients receiving anticoagulant therapy was made at a district general hospital over a two year period. Fifty haemorrhagic incidents occurred as a complication of this therapy, nine in inpatients and 41 in outpatients. At any time during the two years, approximately 12 inpatients and 100 outpatients were receiving anticoagulant therapy. Excessive dosage with anticoagulant was the cause of bleeding in 14 cases; eight patients had pathologic conditions which caused haemorrhage during anticoagulant therapy; drug interaction occurred in seven cases; increased sensitivity to heparin caused bleeding in two cases; in a further two cases multiple factors were responsible. No obvious cause for haemorrhage was found in a further 17 patients. Measures to increase the safety of this form of treatment are discussed, which affect the patient, the clinician and the laboratory directly.

TABLE I

The Clinical Diagnoses of 50 patients who bled during Anticoagulant Therapy

| *Clinical Diagnosis:* | *Number of patients suffering haemorrhage during anticoagulant therapy.* |
|---|---|
| Deep vein thrombosis | 10 |
| Mitral valve disease with systemic embolism | 8 |
| Deep vein thrombosis+pulmonary embolism | 6 |
| Myocardial infarction—prophylactic | 4 |
| Carotid artery stenosis+cerebral embolism | 4 |
| Recurrent deep vein thrombosis | 3 |
| Myocardial infarction+systemic embolism | 2 |
| Mitral valve disease—prophylactic | 2 |
| Cardiac valve prosthesis—prophylactic | 2 |
| Vein grafts—prophylactic | 2 |
| Axillary vein thrombosis | 2 |
| Cardiomyopathy+cerebral embolism | 1 |
| Retinal vein thrombosis | 1 |
| Middle cerebral artery occlusion | 1 |
| Transient cerebral ischaemic attacks | 1 |
| Pulmonary embolism | 1 |
| Total | 50 |

TABLE 2

The distribution of the sites of bleeding in 50 patients during Anticoagulant Therapy

| *Sites of bleeding* | *Number of patients affected* |
|---|---|
| Haematuria | 16 |
| Subcutaneous bruising | 14 |
| Retroperitoneal haemorrhage | 3 |
| Intracranial haemorrhage | 3 |
| Epistaxis | 3 |
| Haematuria+subcutaneous bruising | 2 |
| Gastrointestinal haemorrhage | 2 |
| Subcutaneous bruising+subconjunctival haemorrhage | 1 |
| Gastrointestinal haemorrhage, haemoptysis +possibly haemarthrosis | 1 |
| Intramuscular bleeding plus bleeding gums | 1 |
| Intramuscular bleeding | 1 |
| Bleeding into operation wound | 1 |
| Bleeding into scrotum | 1 |
| Subconjunctival haemorrhage | 1 |
| Total | 50 |

### REFERENCES

ASKEY, J. M., Cherry, C.B. 1950. Thromboembolism associated with auricular fibrillation (Continuous Anticoagulant therapy). **Journal of the American Medical Association, 44**, 97—100.

COON, W. W., Willis, P. W. 1974. Haemorrhagic complications of anticoagulant therapy. **Archives of Internal Medicine, 133**, 386—392.

EASTHAM, R. D. and Morgan, E. H. 1964. Plasma hypercoagulability in patients with carcinoma and after haemorrhage. **Lancet 2**, 543—544.

EASTHAM, R. D. 1968. Improved control of long-term anticoagulant therapy. **British Medical Journal, 2,** 337—340.

EASTHAM, R. D., Perham, T. G. M., Pocock, P. U. (1972) Warfarin and Hydroxyethylrutosides in Deep Vein Thrombosis. **British Medical Journal, 4**, 491—494, 1972.

EASTHAM, R. D. 1972. The prevention of bleeding during long-term oral anticoagulant therapy. **Bristol Medico-Chirurgical Journal, 87**, 5—7.

FULLER, J. A. 1959. Long-term anticoagulant treatment. **Lancet, 2**, 489—492.

HAZARD, J., Mignot, B., Gaubler, J., Domart, A. 1967. A propos de dix haemorrhagies intraraniennes survenues au cours du traitments anticoagulants. **Semaine des hopitaux de Paris, 43**, 2849—2855.

Second Report of the Working Party on Anticoagulant Therapy in Coronary Thrombosis to the Medical Research Council. 1964. An assessment of long-term Anticoagulant Administration after cardiac infarction. **British Medical Journal, 2**, 837—843.

SERRADIMIGNI, A., Armoux, E., Raybaud, M., Giraud, M., Pinas, E., Audier, M. (1969) Les haemorrhages des anticoagulants. **Marseilles Medical, 106**, 1041—1044.

STRAUB, P. W. 1970. Indikationen und Gefahren der Antikoagulantientherapie. **Schweizische Medizinische Woschenschrift, 100**, 686—691.

